# Radiomic-based diagnostics in oncology: challenges toward clinical practice

**DOI:** 10.18632/oncoscience.536

**Published:** 2021-06-02

**Authors:** Emanuele Barabino, Giovanni Rossi, Alessandro Fedeli, Giuseppe Cittadini, Carlo Genova

**Affiliations:** ^1^Interventional Angiography, Ospedale Santa Corona - ASL 2 Savonese, Pietra Ligure, Italy; ^2^Medical Oncology Department, Ospedale Padre Antero Micone, Genova, Italy; ^3^Department of Medical, Surgical and Experimental Sciences, University of Sassari, Sassari, Italy; ^4^Department of Electrical, Electronic, Telecommunications Engineering, and Naval Architecture, University of Genoa, Genova, Italy; ^5^UO Radiologia Generale, IRCCS Ospedale Policlinico San Martino, Genova, Italy; ^6^UOC Clinica di Oncologia Medica, IRCCS Ospedale Policlinico San Martino, Genova, Italy; ^7^Dipartimento di Medicina Interna e Specialità Mediche (DiMI), Facoltà di Medicina e Chirurgia, Università degli Studi di Genova, Genova, Italy

**Keywords:** radiomics, lung cancer, oncology, artificial intelligence, machine learning

Since radiologic exams were converted from analog to digital images, radiologists have made efforts to extract quantitative data from them. In 2012, the word “radiomics” was introduced by Lambin [1] to describe this new science that aims at extracting quantitative features from diagnostic images [2]. One of the possible applications of radiomics in oncology is the investigation of connections between specific radiological phenotypes and molecular status, which in turn translates into personalized therapeutic decisions. Advantages of radiomics are non-invasiveness and the ability to describe pathological processes in their entirety (i.e., the gross tumor volume) and *in-vivo* (e.g., peri-tumoral environment, anatomical relationships), unlike biopsy. The dimensionality of features, the so-called “large p, small n” problem, which is the disproportion between the number of features (p) and images/patients (n) involved in the study, made radiomic features hardly processable with univariate tests, because of the random chance of statistically significant findings [3], or multivariable statistics, due to high correlation between features [4]. Since the amount of information collected through radiomic features makes traditional statistical analyses impractical, they were mostly abandoned in favor of other solutions, such as Artificial Intelligence (AI) and Machine Learning (ML). In order to reduce dimensionality of data but preserve their informative content, prominent roles are played by Principal Component Analysis (PCA) [4] or feature selection by Least Absolute Shrinkage and Selection Operator (LASSO). Notably, ML/AI methods allow creating flexible predictive models, based on few, almost uncorrelated but reliable features. In the first decade of development, radiomic studies were mostly monocentric (or even “mono-scanner”), based on unstandardized features computed by custom in-house software, required manual segmentation, and lacked external validation, resulting in scarcely reproducible models.

In 2021 our group published a study on *EGFR* mutations in NSCLC [5] that started as a monocentric study and consequently evolved during the revision process. We decided to provide a robust initial features selection using a scarcely applied but effective technique, test & re-test. This technique, based on the repetition of a radiological exam to perform cross-reference on data and eliminate radiomic features considered unreliable, in clinical practice clashes with evident ethical issues due to radiation exposure. However, one radiologic exam can provide repeated images: trans-thoracic lung biopsy. Our first predictive ML model achieved 94% accuracy in the internal validation set, but when we tested it on a public dataset available on The Cancer Imaging Archive, accuracy did not exceed 60%. Two problems became evident: our initial model was too dependent on the training cohort and a proper scaling of features was needed to ensure an optimal use of PCA. Once such problems were solved, we realized that diversity within data is not an issue but a value, as an improved variability forces the predictive model to become less dependent on the original cohort and, therefore, more generalizable. To enhance generalization capabilities, we introduced a small dataset from another hospital of our province in the training set. As a consequence, accuracy decreased in our cohort to 88.1% but significantly improved in external test sets (76.6% and 83.3%, respectively), leading to the final predictive model.

Nowadays, researchers are sharing radiological images to create larger, diversified, public datasets to test newly developed predictive models and many efforts have been made to standardize radiomic features and to create a shared language [6]. Manual segmentation has been gradually substituted by automatic or AI-based algorithms, which made the process faster and more reproducible. Still, many questions remain open and need answers before proceeding further: investigators perform radiomic analysis to answer a specific question (mutational status, prediction of response to therapy) but the connection between radiomic features and biological variables is not straightforward in most cases. Moreover, the correct harmonization of radiomic data between different centers still represents a challenge, especially with small and heterogeneous datasets. Another issue that may prevent the translation of radiomic analyses into clinical practice is related to the lack of prospective randomized trials.

Nonetheless, radiomics currently represents the top of exploration into radiological images: as the atom has been, in physics, the smallest analyzable measure of matter for quite a time, so the pixel/voxel represents for radiomics. Soon, using new generation CT, PET and MR scanners with improved signal and higher spatial resolution, we will be able to further reduce pixel dimensions, explore new aspects of the radiological image and, hopefully, get some insights on its biological counterpart. The human body or a tumor are not isolate entities, but complex biological processes affected by multiple variables; considering only a part of those variables will give a limited view of the process in its entirety [7]. An example is the activity of immune checkpoint inhibitors (anti-PD1/L1), which is not determined exclusively by the level of expression of the target (which is itself heterogeneous within the same tumor), but also by other immunological checkpoints, or by the expression of these receptors on other tissues [8], or even on the disposition of lymphocytes in the tumor (inflamed, immuno-excluded and immune-desert) [9] which can compromise the activity of the drug itself. As the most promising aspect of radiomics may reside in the capability of describing the complexity of biological processes, it is, perhaps, the best tool at our disposal to photograph tumor heterogeneity. If we succeed in translating radiomics into clinical practice, we will obtain a tool that can answer to new, more complex questions leading to a factual personalized medicine.
